# Prediction of Gestational Diabetes Mellitus in the First Trimester of Pregnancy Based on Maternal Variables and Pregnancy Biomarkers

**DOI:** 10.3390/nu16010120

**Published:** 2023-12-29

**Authors:** Antigoni Tranidou, Ioannis Tsakiridis, Aikaterini Apostolopoulou, Theodoros Xenidis, Nikolaos Pazaras, Apostolos Mamopoulos, Apostolos Athanasiadis, Michail Chourdakis, Themistoklis Dagklis

**Affiliations:** 1 3rd Department of Obstetrics and Gynecology, School of Medicine, Faculty of Health Sciences, Aristotle University of Thessaloniki, 54642 Thessaloniki, Greece; antigoni.tranidou@gmail.com (A.T.); iotsakir@gmail.com (I.T.); xenidistheodoros@yahoo.com (T.X.); amamop@auth.gr (A.M.); apostolos3435@gmail.com (A.A.); 2 Laboratory of Hygiene, Social & Preventive Medicine and Medical Statistics, School of Medicine, Faculty of Health Sciences, Aristotle University of Thessaloniki, 54124 Thessaloniki, Greece; katapost@yahoo.gr (A.A.); nikpazaras@hotmail.com (N.P.); mhourd@gapps.auth.gr (M.C.)

**Keywords:** gestational diabetes mellitus, GDM, early screening, prediction, first trimester, pregnancy

## Abstract

Gestational diabetes mellitus (GDM) is a significant health concern with adverse outcomes for both pregnant women and their offspring. Recognizing the need for early intervention, this study aimed to develop an early prediction model for GDM risk assessment during the first trimester. Utilizing a prospective cohort of 4917 pregnant women from the Third Department of Obstetrics and Gynecology, Aristotle University of Thessaloniki, Greece, the study sought to combine maternal characteristics, obstetric and medical history, and early pregnancy-specific biomarker concentrations into a predictive tool. The primary objective was to create a series of predictive models that could accurately identify women at high risk for developing GDM, thereby facilitating early and targeted interventions. To this end, maternal age, body mass index (BMI), obstetric and medical history, and biomarker concentrations were analyzed and incorporated into five distinct prediction models. The study’s findings revealed that the models varied in effectiveness, with the most comprehensive model combining maternal characteristics, obstetric and medical history, and biomarkers showing the highest potential for early GDM prediction. The current research provides a foundation for future studies to refine and expand upon the predictive models, aiming for even earlier and more accurate detection methods.

## 1. Introduction

The number of pregnancies diagnosed with gestational diabetes mellitus (GDM) shows a steadily rising trend worldwide, parallel to the increasing maternal age (MA) and prevalence of obesity [1,2]. In addition to the synergistic effect of hormonal changes that occur during pregnancy, risk factors for GDM include higher parity and personal history of previous GDM [3,4]. Results from a meta-analysis report that GDM risk may be decreased by avoiding significant weight gain during pregnancy [5]. In addition, in a recent network meta-analysis, Wu et al. studied overweight and obese individuals. Their findings revealed two effective strategies for limiting gestational weight gain; physical activity alone and physical activity combined with a proper diet. These approaches were especially beneficial for reducing the risk of GDM in the study participants [6]. Moreover, current evidence shows that excessive gestational weight gain among individuals with pre-pregnancy obesity category 2 during the first half of pregnancy is associated with higher odds for GDM occurrence, while, in the normal weight pre-pregnancy individuals, a weight gain beyond 8 kg, in the first trimester of gestation, is associated with higher incidence of GDM [7].

This metabolic dysregulation has been gaining increasing focus as GDM is associated with a higher risk of adverse perinatal outcomes, including preeclampsia, macrosomia, shoulder dystocia, preterm delivery, cesarean delivery and neonatal hyperinsulinemia [8,9]. Furthermore, the risk of future metabolic disorders for both the mother and the infant are increased; they include maternal higher risk for cardiovascular disease, metabolic syndrome, type 2 diabetes mellitus (T2DM), as well as ophthalmic, psychiatric or renal disease. In children, there is increased risk for T2DM, excess adiposity, impaired neurodevelopment, neuropsychiatric, and ophthalmic disease [10,11,12].

The state of the art in GDM prediction has evolved significantly over recent years. Advanced statistical and machine learning models have been increasingly applied to improve the accuracy and timeliness of GDM prediction [13]. Despite these advancements, the heterogeneity in populations studied, differences in diagnostic criteria, and variability in predictor variables used across studies pose challenges in standardizing and adopting these models universally [14]. Concurrently, systematic reviews focusing on routine screening methods have highlighted the importance of traditional risk factors and clinical markers in predicting GDM. These studies underscore the effectiveness of integrating clinical data from routine antenatal care with prediction models to enhance early detection and management of GDM [15].

Notably, screening for GDM differs across national and international guidelines and depends on preexisting risk factors [16]. It is not uncommon that metabolic dysregulation may be present prior to conception; the underlying insulin resistance increases the odds for developing GDM in these women [17]. Accordingly, some women may experience GDM earlier in the course of their pregnancy, especially in the presence of aggravating factors such as excess adipose tissue [18]. Hence, regardless of the onset of metabolic alterations, it is critical for pregnant women to undergo prenatal care that allows timely identification of GDM. Towards this end, a number of studies have implemented early prediction models based on various biometric indicators like adiponectin/leptin ratio [19], inflammation markers like C-reactive protein (CRP) [20], as well as anthropometric parameters, e.g., age, weight, obstetric history [21], with each of the models achieving variable degrees of predictability, probably attributable to the different ethnic groups, the data available for each study group, the diversity of adjusted factors and various diagnostic standards.

The significance of computing the risk associated with GDM at early stages of pregnancy is highlighted, as timely intervention may be crucial for the prevention of adverse pregnancy outcomes. Thus, the aim of this study was to assess the risk factors and create a prediction model for GDM in the first trimester of pregnancy.

## 2. Materials and Methods

The data used in this study are prospectively (August 2020–December 2022) collected as part of an ongoing cohort study on diabetes in pregnancy, named “BORN2020”. All attendees were recruited at the Third Department of Obstetrics and Gynecology, School of Medicine, Faculty of Health Sciences, Aristotle University of Thessaloniki, Greece. Women were recruited at their first trimester antenatal visit at 11^+0^–13^+6^ weeks of gestation. Eligibility criteria were the following: (i) age > 18 years old, (ii) absence of preexisting diabetes (type 1 or 2), (iii) pregnancies that did not end as miscarriage or termination before 28 weeks of gestation; this criterion was applied to ensure inclusion of only those pregnancies where GDM screening was viable and relevant, and (iv) pregnancies continuing beyond 28 weeks of gestation, to ensure that GDM screening was performed. A total of 4532 women were initially recruited. Of these, 335 women were subsequently excluded due to miscarriage or termination before 28 weeks of gestation. This exclusion was necessary to focus our analysis on viable pregnancies where GDM screening is typically performed. Therefore, the final cohort for the study comprised 4917 women.

Participants’ data included the following information: maternal age (MA), body mass index (BMI) before pregnancy, smoking status, method of conception, obstetric history and history of thyroid disease or chronic hypertension. Chronic hypertension was identified based on participants’ self-reported medical history, validated by medical records, and defined as blood pressure ≥ 140/90 before the 20th week of pregnancy. Furthermore, serum PAPP-A and free β-hCG were assessed as part of routine screening for Down’s syndrome. Mean uterine artery pulsatility index (UtA-PI) was measured by transabdominal pulsed wave Doppler ultrasonography, as part of routine screening for preeclampsia.

With regards to GDM screening, the Hellenic Society of Obstetricians and Gynecologists recommends universal screening at 24–28 weeks of gestation, using GDM diagnostic criteria that are based on the results of the “Hyperglycemia and Adverse Pregnancy Outcome” (HAPO) study [22]. Specifically, after fasting for at least 8 h, women are tested for glucose levels and then they are given 75 g of oral glucose and tested again at one and two hours. GDM is diagnosed when at least one of the following cutoffs is met: fasting blood glucose ≥ 92 mg/dL, blood glucose concentration 1 h after OGTT ≥ 180 mg/dL and/or blood glucose concentration 2 h post OGTT ≥ 153 mg/dL.

The study received approval by the Bioethics Committee of Aristotle University of Thessaloniki, Greece (6.231/29 July 2020). Written consent was obtained from all participants.

### Statistical Analysis

Regarding univariate feature analyses of maternal characteristics, medical and obstetric history and measured variables, the following methods were applied: in case of continuous variables, the Shapiro–Wilk test was used to determine normality, the F-test was used to determine variance equality, and the t-test, Wilcoxon test, or Mann–Whitney test was used to test the validity of the hypothesis. For binary variables the Fisher’s exact test was applied.

Values of fβ-hCG and PAPP-A were converted into multiples of the median (MoM). The UtA-PI was expressed as a z-score. The correlation between maternal characteristics, obstetric history, pregnancy biomarkers and the probability of subsequently developing GDM was examined using multivariate logistic regression analysis. Five models were tested to detect GDM early. The models were created based on data readily available in routine care practice. The features of each model were selected based on clinical characteristics proposed in previous studies [21,23,24], and thus, different categories were formed by the available data (maternal characteristics, obstetric history, biomarkers and measured variables) with regards to known risk factors for GDM development. An addition to our models, not extensively reported in the previous literature, was the computation of fβ-hCG. The detection rates at fixed false-positive rates (FPR) of 5%, 10%, 15%, and 20% were provided, as well as the area under the ROC curve (AUROC). Bootstrapping was used to compute confidence intervals for the detection rate of each model, given the specific fixed false positive rate. The differences in AUROC across the five prediction models were evaluated. The R programming language was used for all statistical implementations (v4.2.1).

## 3. Results

Overall, 4917 women were eligible based on the study selection criteria. Of these, 474 (9.64%) developed GDM and 4443 women (90.4%) did not. The median MA of the GDM population was higher in the GDM group compared to the non-GDM group (*p* < 0.0001), while 39% (n = 183) of women with GDM and 26% (n = 1138) of women without GDM were >35 years old (*p* < 0.0001). Pre-pregnancy BMI was higher in the GDM group vs. the non-GDM group (*p* < 0.0001). Moreover, conception via ART was higher in the GDM group (*p* < 0.0001), and smoking during pregnancy was also higher in the GDM group (*p* = 0.03). In addition, chronic hypertension (*p* = 0.02), thyroid disease (*p* = 0.01), history of preeclampsia (*p* = 0.003) and history of previous cesarean section (*p* = 0.001) were higher in the GDM women compared to the non-GDM individuals. Finally, PAPP-A levels (*p* = 0.04) and also the UtA-PI z-score (*p* = 0.03) were lower in the GDM individuals. Maternal characteristics and available biomarkers are presented in Table 1.

In the multivariate analysis, as shown in Table 2, several models were evaluated for their ability to predict GDM:

Model 1: MA and pre-pregnancy BMI were independent contributors for GDM (aOR: 1.08; 95% CI: 1.05–1.09; *p* < 0.0001 and aOR: 1.10; 95% CI: 1.08–1.12; *p* < 0.0001, respectively).

Model 2: Similar to Model 1, MA and pre-pregnancy BMI were associated with higher incidence of GDM (aOR: 1.07; 95% CI: 1.04–1.09; *p* < 0.0001 and aOR: 1.10; 95% CI: 1.08–1.12; *p* < 0.0001, respectively).

Model 3: Added history of preeclampsia to the factors in Model 2, identifying it as an independent contributing factor for GDM (aOR: 1.80; 95% CI: 1.12–2.81; *p* = 0.01), while higher parity was associated with reduced incidence of GDM (aOR: 0.72: 95% CI: 0.56–0.92; *p* = 0.009).

Model 4: Included MA (aOR: 1.08; 95% CI: 1.06–1.10; *p* < 0.0001), levels of PAPP-A (aOR: 0.83; 95% CI: 0.70–0.98; *p* = 0.03) and UtA-PI (aOR: 0.89; 95% CI: 0.80–0.98; *p* = 0.02) were independent contributors for GDM.

Model 5: the combination of all the available data showed that MA, pre-pregnancy BMI and history of preeclampsia were strongly correlated with increased risk GDM (aOR: 1.07; 95% CI: 1.05–1.09; *p* < 0.0001, aOR: 1.10; 95% CI: 1.08–1.12; *p* < 0.0001 and aOR: 1.78; 95% CI: 1.11–2.80; *p* = 0.01, respectively), while higher parity was found to be associated with reduced rates of GDM (aOR: 0.73; 95% CI: 0.57–0.85; *p* = 0.01).

### Model Performance and Comparison

As shown in Table 3, the AUROCs of each model was used to assess performance:

Model 1: AUROC of 0.672, using maternal characteristics and smoking.

Model 2: Similar to AUROC of Model 2 that used maternal characteristics and maternal medical history (0.672).

Model 3: Slight improvement of the AUROC (0.675) with the use of obstetric history.

Model 4: Lower predictability when pregnancy biomarkers were employed (AUROC of 0.606).

Model 5: Slightly higher AUROC (0.678) compared to other models.

**Figure 1:**
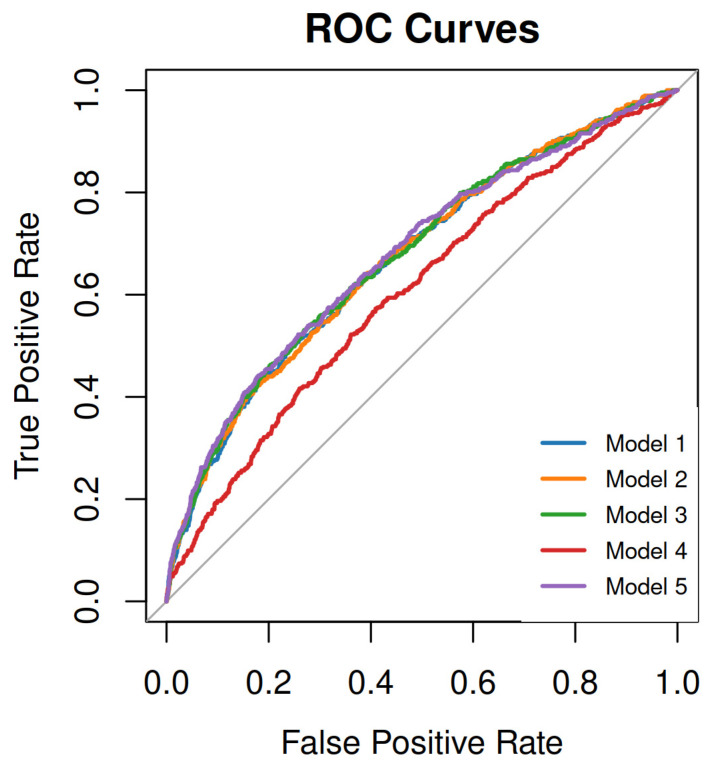
ROC curves of the 5 models for early screening of GDM. Model 1: maternal characteristics (maternal age, BMI before pregnancy), smoking during pregnancy; Model 2: maternal characteristics, maternal medical history (thyroid disease, chronic hypertension conception via assisted reproductive technology; Model 3: maternal characteristics, obstetric history (history of preeclampsia, small for gestational age neonate, previous cesarean delivery, parity); Model 4: maternal characteristics, pregnancy biomarkers (free-beta subunit human chorionic gonadotropin, plasma protein-A) and measured variables (uterine artery pulsatility index); Model 5 is a comprehensive model combining all elements of the previous models, including maternal characteristics, smoking during pregnancy, maternal medical history, conception with assisted reproductive technologies, obstetric history, and pregnancy biomarkers.

Models 1, 2, 3, and 5 have similar performance and the difference of their AUROCs is not statistically significant. Model 4 is the only model that is statistically different from Models 1, 2, 3, and 5. In Table 4, the detection rates for all models at 5%, 10%, 15% and 20% fixed FPR are presented, while Figure 1 presents the ROC curves of the five models that were employed.

## 4. Discussion

Results from this study showed the following: (i) The incidence of GDM in our population was 9.6%; (ii) In the first trimester of pregnancy, for the models combining maternal characteristics with obstetric history (i.e., Models 3 and 5), the following variables were independent risk factors for GDM: maternal age, pre-pregnancy BMI and history of preeclampsia. This underscores the importance of these factors in early intervention strategies. Mirabelli et al. further emphasize the significant impact of maternal preconception BMI over age as a risk factor for GDM, suggesting that interventions targeting preconception weight management might be particularly effective in areas with high GDM prevalence [25]; (iii) the predictive model combining maternal characteristics (MA, pre-pregnancy BMI), maternal medical and obstetric history, as well as pregnancy biomarkers, for early GDM screening (Model 5) had the highest predictive value. This finding highlights the potential of integrated models in improving early detection and management of GDM, which is crucial for reducing associated risks; Lastly, (iv) the inclusion of fβ-hCG in the multivariate model for GDM prediction (Model 4) had the lowest performance compared to the other models. This result indicates that not all biomarkers are equally predictive for GDM, emphasizing the need for critical selection and combination of variables in model development.

The variations between the national and international recommendations on screening for GDM were recently reviewed in a comparative analysis by Tsakiridis et al. [16]. In particular, the American College of Obstetricians and Gynecologists (ACOG), as well as the Australasian Diabetes in Pregnancy Society (ADIPS), the Society of Obstetricians and Gynecologists of Canada (SOGC), and the International Federation of Gynecology and Obstetrics (FIGO) suggest screening at 24–28 weeks of gestation for all pregnant women that have no other risk factors, while in contrast, the National Institute for Health and Care Excellence (NICE) recommends screening at 24–28 weeks only for those who have risk factors. The Endocrine Society (ES) recommends universal screening at the beginning of the first trimester for all pregnant women and if negative, repeat testing at 24–28 weeks of gestation. The FIGO guideline recommends this latter approach of screening only in nations with a higher risk of developing GDM.

In a systematic review, the authors identified the constraints of the available performance models implemented for the prediction of GDM [26]. The biomarkers addressed for the prediction of GDM demonstrate that their predictive ability is constrained, and conflicting results have been reported. Moreover, studies on the added value of readily available biomarkers to noninvasive models are rare. Another meta-analysis highlighted the need for implementing more sophisticated risk prediction algorithms and more research on precise (bio)markers [15]. Our study aimed to identify possible markers for early GDM prediction, with the use of different combinations of the available biomarkers and non-invasive measures as compared to those referred in literature. Data were collected during routine care screening, while the population included participants with and without known risk factors for GDM.

The incidence of GDM in our population is consistent with previous European studies [27,28]. In addition, a recent meta-analysis indicated that the overall weighted prevalence of GDM in a total of 24 European countries was 10.9% [29]. The highest prevalence of GDM was observed in Eastern Europe (31.5%), while the lowest was recorded in Northern Europe with 8.9%. The corresponding values for Western and Southern Europe were 10.7% and 12.3%, respectively.

Several risk factors have been identified for the development of GDM. In our study, GDM was associated with higher MA and increased pre-pregnancy BMI, as has been described in previous studies [30,31]. Yong et al. reported that in women of older age that started their pregnancy while being overweight or obese, the risk for GDM increased by 2.5 times, while gaining excess weight during pregnancy aggravated these odds [31]. Moreover, in a study conducted by Buerger et al., the authors evaluated the risk for GDM in singleton and twin pregnancies [32]. The odds were higher with advancing MA, increased weight and birthweight z-score from previous pregnancy, in both cases. The risk remained higher in cases where there was personal history of GDM or for individuals with a first- or second-degree family history of diabetes and for women that conceived with the use of medications for ovulation induction. The authors applied this information in the development of a screening model for GDM; therefore, setting the FPR at 10% and 20% for singleton pregnancies, the detection rates were 42.8% and 58%, respectively. The screening accuracy of the model by Buerger et al. may be overestimated as the authors declare that their large sized control group may have included women with undiagnosed GDM, as OGTT was not applied in all pregnancies [32]. Likewise, in a predictive model used by Shen at al., which computed the maternal characteristics and obstetric history of pregnant women in screening for GDM, at FPR of 10% and 20%, the detection rates were 35.6% and 53%, respectively [21]. Moreover, when the authors compared maternal characteristics, obstetric history and preeclampsia biomarkers, the AUROC of the model yielded more accuracy (0.738). But as mentioned by the authors, the results may be biased towards underestimating the risk for GDM because the OGTT screening was based on a targeted screening strategy. The FPR at 10% and 20% in this case yielded detection rates at 36.7% and 51.5%, respectively.

Furthermore, the use of UtA-PI has been previously studied. More specifically, Chatzakis et al. investigated the phenotype subgroups of GDM in relation to preeclampsia and UtA-PI percentiles and reported significant differences among the three distinguished phenotypes [33]. The first phenotype included individuals with abnormal fasting blood glucose levels, the second included women with abnormal blood glucose levels after one or two hours following the OGGT, while the third phenotype included a combination of the other two phenotypes. The third phenotype was associated with a higher uterine artery resistance and higher incidence of preeclampsia. The correlation between GDM and preeclampsia is well established in the literature [34]. In our study, the UtA-PI was associated with increased risk for GDM. However, the model that used this index achieved the lowest predictability. Furthermore, in a meta-analysis by Talasaz et al., the prognostic value of plasma protein-A (PAPP-A) was found of low predictive value when used alone. However, if used collectively with other tests, it may have a better prognostic result [35]. In the current study, lower levels of PAPP-A were detected in the GDM population in comparison to the non-GDM individuals. The incorporation of PAPP-A in Model 4, which combined maternal characteristics and measured variables showed that low levels of PAPP-A were associated with 17% lower chance for GDM incidence (aOR: 0.83, 95% CI [0.70, 0.98], *p* = 0.03). As per our study findings, the incidence of previous cesarean delivery was associated with GDM. This finding is in accordance with previous studies [36]. Particularly, according to Xiong et al., among other risk factors for GDM, prior cesarean section increased the risk for GDM by 18% [37].

The strengths of this study include the representative sample size, the prospective systematic method to collect obstetric and medical history, the implementation of universal GDM screening with the same diagnostic criteria and the implementation of biomarkers (fβ-hCG, PAPP-a and UtA-PI) in the model to assess its use for the early diagnosis of those at risk for developing GDM. One of the limitations of our study is that we did not have consistent information on family history of GDM or GDM occurrence in previous pregnancy which are important predictors for GDM and this may have underestimated the accuracy of our prediction models. Moreover, all data were collected from a single center and thus, the prediction models lack external verification. As a future work we plan to gather data in collaboration with other settings, to confirm the accuracy of the prediction models implemented in this study.

## 5. Conclusions

In conclusion, our study has demonstrated the utility of comprehensive models in early GDM screening, with Model 5 showing the highest predictability. These findings are significant for clinical practice, offering a potential pathway for early intervention and improved outcomes. However, the study also highlights the complexity of the early prediction of GDM and the need for ongoing research. Future research should focus on validating these models in diverse populations and settings to enhance their generalizability and clinical utility. Additionally, the exploration of new biomarkers and risk factors, as well as the application of advanced statistical and machine learning techniques, could further refine GDM prediction models. The main goal is to develop a universally applicable, non-invasive, and accurate prediction model that can be readily implemented in the early course of pregnancy.

By critically reflecting on the study’s findings and outlining a clear path for future research, this study contributes to the evolving landscape of GDM prediction and underscores the importance of continued innovation and critical evaluation in this field.

## Figures and Tables

**Table 1:** Maternal characteristics of women with and without gestational diabetes.

Maternal Characteristics	GDM (N = 474)	Non-GDM (N = 4443)	*p* Value
MA (years) 25%, 50%, 75%	33.5 30, 33.5, 36.9	31.8 28.4, 31.8, 35	<0.0001
MA > 35 (n%)	183 (38.6)	1138 (25.6)	<0.0001
BMI pre (kg/m^2^)	25 22.2, 25, 30.5	22.7 20.7, 22.7, 25.7	<0.0001
Conception via ART (n%)	44 (9.3)	209 (4.7)	<0.0001
Smoking during pregnancy (n%)	67 (14.1)	481 (10.8)	0.03
Chronic Hypertension (n%)	5 (1.05)	12 (0.27)	0.02
Thyroid disease (n%)	55 (11.6)	356 (8.01)	0.01
SLE/APS (n%)	3 (0.63)	23 (0.51)	0.73
Obstetric history			
Preeclampsia (n%)	28 (5.9)	136 (3.06)	0.003
SGA (n%)	2 (0.42)	34 (0.76)	0.57
PCS (n%)	105 (22.2)	719 (16.2)	0.001
Parity (n%)	202 (42.6)	1807 (40.7)	0.43
Measured variables			
fβ-hCG MoM	0.46, 0.9, 1.27	0.65, 0.97, 1.47	0.11
PAPP-A MoM	0.9, 1.07, 1.26	0.94, 1.09, 1.26	0.04
UtA-PI z-score	−0.50, 0.20, 0.88	−0.24, 0.28, 0.88	0.03

BMI pre: Body Mass Index before pregnancy; Conception ART: conception with assisted reproductive technologies; fβ-hCG: free-beta subunit human chorionic gonadotropin; GDM: gestational diabetes mellitus; PAPP-A: plasma protein-A; PCS: previous caesarian section; Thyroid disease: Hypothyroidism, Hyperthyroidism, Hashimoto’s disease; SLE/APS: Systemic lupus erythematosus/Antiphospholipid syndrome; SGA: small for gestational age; UtA-PI: uterine artery pulsatility index.

**Table 2:** Maternal characteristics, maternal medical and obstetric history and measured variables of present pregnancy associated with gestational diabetes.

	Multivariate Regression Analysis, aOR, 95% CI									
	Model 1	*p* Value	Model 2	*p* Value	Model 3	*p* Value	Model 4	*p* Value	Model 5	*p* Value
MA (years)	1.08 (1.05, 1.09)	<0.0001	1.07 (1.04, 1.09)	<0.0001	1.07 (1.05, 1.10)	<0.0001	1.08 (1.06, 1.10)	<0.0001	1.07 (1.05, 1.09)	<0.0001
BMI pre	1.10 (1.08, 1.12)	<0.001	1.10 (1.08, 1.12)	<0.0001	1.10 (1.08, 1.12)	<0.0001			1.10 (1.08, 1.12)	<0.0001
Conception ART			1.44 (0.98, 2.07)	0.052					1.36 (0.91, 1.10)	0.11
Smoking during pregnancy	1.23 (0.92, 1.63)	0.15							1.27 (0.94, 1.70)	0.10
Thyroid disease			1.25 (0.90, 1.69)	0.16					1.25 (0.90, 1.70)	0.15
Chronic hypertension			1.64 (0.49, 4.75)	0.38					1.32 (0.40, 3.91)	0.62
SLE/APS			1.20 (0.28, 3.54)	0.76					1.14 (0.27, 3.40)	0.83
Preeclampsia History					1.80 (1.12, 2.81)	0.01			1.78 (1.11, 2.80)	0.01
SGA History					0.49 (0.08, 1.68)	0.33			0.51 (0.08, 1.78)	0.37
PCS History					1.24 (0.91, 1.67)	0.16			1.21 (0.89, 1.64)	0.21
Parity					0.72 (0.56, 0.92)	0.009			0.73 (0.57, 0.85)	0.01
fβ-hCG MoM							0.95 (0.83, 1.07)	0.41	0.97 (0.86, 1.09)	0.63
PAPP-A MoM							0.83 (0.70, 0.98)	0.03	0.86 (0.72, 1.01)	0.07
UtA-PI z-score							0.89 (0.80, 0.98)	0.02	0.94 (0.85, 1.04)	0.22

aOR: adjusted odds ratio; CI: confidence interval; MA: maternal age; BMI pre: Body Mass Index before conception; Conception ART: conception with assisted reproductive technologies; Thyroid disease: Hypothyroidism, Hyperthyroidism, Hashimoto’s disease; SLE/APS: Systemic lupus erythematosus/Antiphospholipid syndrome; SGA: small for gestational age; PCS: previous caesarian section; MoM: multiples of the median; fβ-hCG: free-beta subunit human chorionic gonadotropin; PAPP-A: plasma protein-A; UtA-PI: uterine artery pulsatility index.

**Table 3:** Comparison of prediction models for gestational diabetes. Each cell in the Model Comparison section presents the difference of AUROCs between the model in the row and the model in the column, along with the 95% CI for this difference.

			Model Comparison—Difference of AUROCs (95% CI for the Difference)				
Screening Model for GDM	AUROC (95% CI)	SE	Model 1	Model 2	Model 3	Model 4	Model 5
Model 1	0.672 (0.65–0.70)	0.0141	-	−0.0005 (−0.006, 0.004)	−0.003 (−0.01, 0.006)	0.07 (0.04, 0.09) *	−0.006 (−0.02, 0.004)
Model 2	0.672 (0.65–0.70)	0.0141	-	-	−0.003 (−0.01, 0.007)	0.07 (0.04, 0.09) *	−0.006 (−0.02, 0.004)
Model 3	0.675 (0.65–0.70)	0.0141	-	-	-	0.07 (0.04, 0.09) *	−0.003 (−0.01, 0.004)
Model 4	0.606 (0.58–0.63)	0.0143	-	-	-	-	−0.07 (−0.10, −0.05) *
Model 5	0.678 (0.65–0.70)	0.0140	-	-	-	-	-

AUROC: area under the curve; SE: standard errors; Model 1: maternal characteristics (maternal age, BMI before pregnancy), smoking during pregnancy; Model 2: maternal characteristics, maternal medical history (thyroid disease, chronic hypertension conception via assisted reproductive technology); Model 3: maternal characteristics, obstetric history (history of preeclampsia, small for gestational age neonate, previous cesarean delivery, parity); Model 4: maternal characteristics, pregnancy biomarkers (free-beta subunit human chorionic gonadotropin, plasma protein-A) and measured variables (uterine artery pulsatility index); Model 5 is a comprehensive model combining all elements of the previous models, including maternal characteristics, smoking during pregnancy, maternal medical history, conception with assisted reproductive technologies, obstetric history, and pregnancy biomarkers. * *p* < 0.05 for the difference in AUROC comparison between the models.

**Table 4:** Detection and fixed false positive rates for the predictive models for gestational diabetes.

Detection Rate	Fixed False Positive Rate			
	5%	10%	15%	20%
Model 1	12.26 (5.45, 21.18)	23.79 (14.98, 34.35)	33.04 (24.55, 43.69)	39.1 (29.57, 48.70)
Model 2	11.45 (5.38, 22.01)	24.38 (15.39, 34.5)	32.97 (23.61, 44.5)	39.5 (29.91, 49.81)
Model 3	11.32 (6.03, 20.84)	22.01 (12.64, 32.07)	33.83 (24.53, 43.69)	41.82 (32.72, 51.21)
Model 4	10.65 (4.09, 21.36)	17.58 (9.84, 27.5)	23.54 (14.13, 35.11)	31.71 (20.77, 42.77)
Model 5	12.24 (7.04, 23.46)	20.19 (11.1, 31.51)	30.68 (21.99,41.67)	41.12 (33.24, 50.8)

Model 1: maternal characteristics, smoking during pregnancy; Model 2: maternal characteristics, maternal medical history, conception with assisted reproductive technologies; Model 3: maternal characteristics, obstetric history; Model 4: maternal characteristics, pregnancy biomarkers; Model 5 is a comprehensive model combining all elements of the previous models, including maternal characteristics, smoking during pregnancy, maternal medical history, conception with assisted reproductive technologies, obstetric history, and pregnancy biomarkers.

## Data Availability

The data presented in this study are not publicly available due to privacy restrictions.
